# Walking pace is a protective factor for rheumatoid arthritis: a mendelian randomization study

**DOI:** 10.1038/s41598-024-76666-6

**Published:** 2024-10-22

**Authors:** Qin Zhang, Xiaoxiong Huang, Yazhong Zhang, Zhujun Chao, Ruoran Zhou, Roslida Abd Hamid, Yunfang Zhen, Yusheng Li, Cheng Huang, Wu Xu, Jun Lin

**Affiliations:** 1 Department of Orthopaedics, The Fourth Affiliated Hospital of Soochow University, Suzhou Dushu Lake Hospital, Medical Center of Soochow University, Suzhou, 215000 Jiangsu P.R. China; 2 Department of Orthopaedics, First Affiliated Hospital of Soochow University, Soochow University, Suzhou, 215000 Jiangsu P.R. China; 3 Department of Orthopaedics, Ningbo No. 2 Hospital, Ningbo, 315000 Zhejiang P.R. China; 4 Department of Orthopaedics, The Second Affiliated Hospital of XuZhou Medical University, Xuzhou, 221000 Jiangsu P.R. China; 5 Medical college, Soochow University, Suzhou, 215006 Jiangsu P.R. China; 6 Department of Biomedical Science, Faculty of Medicine and Health Sciences, Universiti Putra Malaysia, Serdang, 43400 Selangor Malaysia; 7 Department of Orthopaedics, Children’s hospital of Soochow University, Suzhou, 215000 Jiangsu P.R. China; 8 Deparment of Orthopaedics, Xiangya Hospital, Central South University, Changsha, 410008 Hunan P.R. China; 9 Deparment of Orthopaedics, China-Japan Friendship Hospital, Beijing, P.R. China

**Keywords:** Walking pace, Rheumatoid arthritis, Mendelian randomization, Causal association, MR-BMA, Computational biology and bioinformatics, Rheumatology

## Abstract

Walking pace is a simple and functional form of exercise and a strong predictor of health, but little is known about its causal association with rheumatoid arthritis. This study aimed to investigate the causal effect of WP on the developing RA using Mendelian randomization analysis. The genetic variation associated with WP was selected as an instrumental variable from the latest genome-wide association studies. Summary-level data for the outcomes were obtained from the corresponding GWAS. The inverse-variance weighted method was used as the primary MR analysis. The results were further tested using a multivariable MR approach based on Bayesian model averaging. Confounders (BMI, SMK, HBP, TD) with close associations with RA were included in the analysis. An observational study with individual data from UK Biobank was performed to reinforce our findings. The MR results indicated the significant inverse associations of WP with the risk of RA (odds ratio (OR), 0.31; 95% confidence interval (CI), 0.15, 0.62; p = 1.05 × 10 −3). After adjusting for the risk factors, the associations for WP and RA did not change substantially. Observational study results demonstrated the same effect of WP on reducing the risk of RA. The Mendelian randomization analysis and observational study provide evidence suggesting that walking pace is a protective factor for rheumatoid arthritis. Given its simple measurement, walking pace may be a pragmatic target for interventions.

**Supplementary Information:**

The online version contains supplementary material available at 10.1038/s41598-024-76666-6.

## Introduction

Rheumatoid arthritis (RA) is a chronic autoimmune disorder characterized by persistent inflammation of the synovial joints, leading to joint pain, swelling, and deformity^1,2^. It affects approximately 1% of the global population and is more prevalent in women than men^3^. RA is associated with significant disability, reduced quality of life, and increased mortality, making it a substantial burden on individuals and healthcare systems worldwide^4^. The pathogenesis of RA involves a complex interplay of genetic, environmental, and immunological factors^5^. Genetic predisposition, particularly the involvement of human leukocyte antigen (HLA) genes, plays a significant role in disease susceptibility. However, environmental factors, such as smoking, obesity, and lifestyle behaviors, also contribute to the development and progression of RA^6–8^.

Walking is a fundamental human movement and a common form of physical activity that is accessible to individuals of all ages and fitness levels^9,10^. Walking pace, defined as the speed at which an individual walks, has gained attention as a simple and objective measure of physical function and overall health^11^. It has been recognized as a valuable indicator of cardiovascular fitness, mortality risk, and various health outcomes^12,13^. Research has shown that walking pace is associated with a range of health measures and outcomes. For instance, a faster walking pace has been linked to better cardiovascular health, including lower blood pressure, improved lipid profiles, and reduced risk of cardiovascular events^14,15^. Additionally, walking pace is a predictor of overall mortality, with slower walking speeds associated with increased mortality risk^16–18^. While the association between walking pace and various health outcomes has been extensively studied, its relationship with specific diseases, such as rheumatoid arthritis (RA), remains an area of interest. Identifying modifiable lifestyle factors that may influence the risk of developing RA is important for preventive strategies and improving patient outcomes.

Mendelian randomization (MR) is a powerful analytical approach that utilizes genetic variants as instrumental variables (IV) to investigate causal relationships between modifiable exposures and disease outcomes. It leverages the random assortment of genetic variants during meiosis, making it less prone to confounding and reverse causation biases compared to traditional observational studies^19–21^. In this study, we aim to employ MR analysis to investigate the potential causal association between walking pace and the risk of developing RA. By utilizing genetic instrumental variables, we can provide robust evidence regarding the potential role of walking pace in RA development, helping inform preventive strategies and interventions for individuals at risk of this debilitating autoimmune disease.

## Method

### Study design

**Fig. 1:**
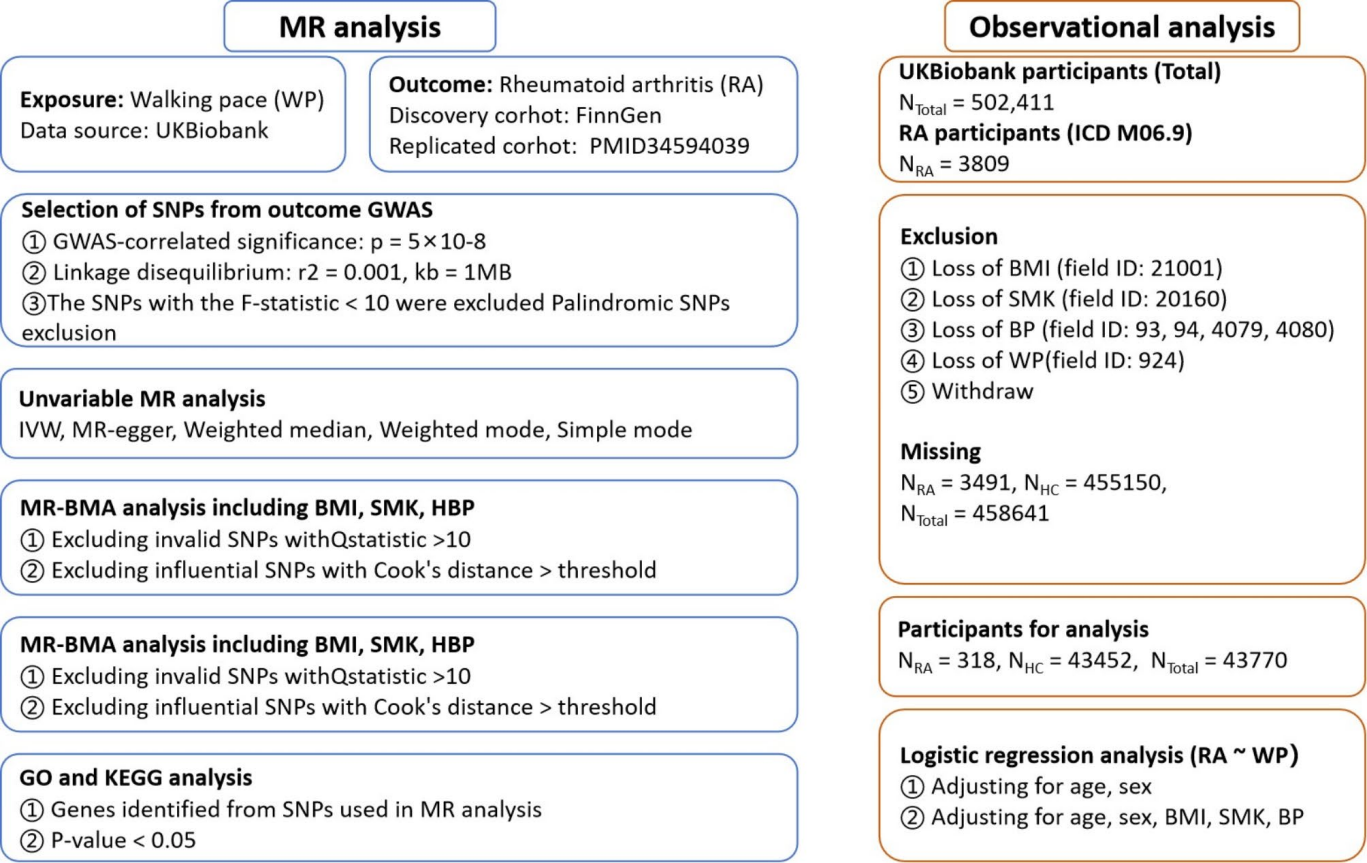
Workflow of the Mendelian randomization and observational analysis.

As shown in the flow chart in Fig. 1, We first extracted SNPs associated with WP from the GWAS data of 459,915 European individuals. In order to verify the correctness of the conclusion, another cohort with Ncase = 8255 and Ncontrol = 409,001 were chosen for repeat validation^22^. Then, using phenoscanner to identify and exclude confounding factors related to RA (*p* < 5 × 10^−8^), and selected the remaining SNPs that overlapped with RA-associated SNPs for the MR analysis^23^. Furthermore, to further validate the results of the MR analysis, we included other causal factors (BMI, SMK, HBP, TD) associated with RA and performed the MR-BMA to examine whether the causal relationship between WP and RA remained after adjusting for these factors^24^. Finally, we utilized individual data of WP and RA from the UK Biobank database, initially adjusting for gender and age, and subsequently further adjusting for the aforementioned risk factors to enhance the reliability of our findings.

### Data source

The characteristics of the data sources in this study are presented in **Supplementary Table ****S1**. No additional ethical approval was required for the use of this database. The current study utilized publicly available summary-level data of WP obtained from the UK Biobank database of 459,915 European individuals. The replicated MR analysis were conducted by another cohort with Ncase = 8255 and Ncontrol = 409,001 obtained from Sakaue, et al.^22^. GWAS data of RA was obtained from a publicly available open-GWAS website (https://gwas.mrcieu.ac.uk/), which was from Finngen, comprising 153,457 participants of European ancestries. The data of confounders (BMI, SMK, HBP, TD) with close associations with RA were all obtained from UK Biobank databases, providing 1,845,984 participants of European ancestries.

### Selection of genetic instruments

The necessary instrumental variables (IVs) were obtained through an initial screening of the related single nucleotide polymorphisms (SNPs). Genetic instruments were selected based on two criteria: (i) *P*_*value*_ of 5 × 10^−8^ and (ii) Clumping r^2^ of 0.001, and clumping distance cutoff < 10,000 kb. Phenoscanner were used to determine the trait of SNPs under the setting of *P*_*value*_ = 5 × 10^−8^, r^2^ = 0.8, and SNPs whose trait contain inflammation and inflammatory cells were removed. Moreover, the F-statistics for each SNP were calculated to assess the effect of intensity of the IVs, to minimize potential weak instrument bias, SNPs with F-statistics < 10 were excluded^25^. The screening flowsheet is shown in Fig. 1. The exposure and outcome data were harmonized to ensure that the effect of a SNP on the exposure and the effect of that SNP on the outcome correspond to the same allele. All information about harmonized SNPs is shown in **Supplementary Table S2**.

### Unvariable MR estimates

The R software was utilized to estimate MR results through the application of “TwoSampleMR” packages. Effect estimates were reported in odds ratios (ORs) values when the outcome was dichotomous. Phenoscanner was used to detect and remove cofounder SNPs associated. IVW was utilized as the primary outcome, while MR-Egger, weighted mode, weighted median, and Simple mode enhanced the IVW estimates by generating more robust estimates in a wider range of circumstances^26,27^. The MR Egger test was employed to assess the pleiotropy effect, and Cochran’s Q test was applied to identify heterogeneity^28^. The flow diagram is illustrated in Fig. 1.

### MR-BMA estimates

To overcome the limitations of traditional logistic regression methods and further validate the reliability of the results, we applied a novel analysis method based on Bayesian Model Averaging (BMA)^24^. The effectiveness of instruments in the model was examined using the Q-statistic. The Cook’s distance was used to quantify influential genetic variations^29^.

### Observational analysis

UK Biobank was used for observational analysis. The project number was 80,532. RA participants was defined by using ICD codes (M06.9). The participants with full information including, BMI (field ID: 21001), ever smoked (field ID: 20160), blood pressure (field ID: 93, 94, 4079, 4080), and usual walking pace (field ID: 924) was included in this analysis. Finally, participants of European individuals in the UK Biobank (*n* = 43,770) including the RA group (*n* = 318) is presented in the **Supplementary Table S3**. According to the population characteristics provided, the logistic regression analysis was initially conducted, adjusting solely for age and gender. Subsequently, a multiple regression analysis was performed, incorporating other risk factors including BMI, SMK, and HBP.

### Gene ontology (GO) and Kyoto Encyclopedia of genes and genomes (KEGG) pathwayenrichment analysis

Phenoscanner was employed to identify the genes where the SNPs, obtained by intersecting the summary data of WP and RA, were located for conducting the MR analysis. To further explore the biological role of WP on the development of RA, GO enrichment analysis was conducted on the identified genes. GO enrichment analysis has been used to annotate genes, gene products, and sequences^30^. GO terms are classified into three categories: cellular components, molecular functions, and biological processes. The KEGG database serves as a repository for the systematic analysis of gene function in numerous biochemical pathways^31^. The Database for Annotation, Visualization, and Integrated Discovery (DAVID) is a web-accessible program for classifying functionally associated genes into a manageable number of biological modules, followed by a systematic analysis of gene modules in a biological context^32^.

### Software and statistics

All analysis were performed in R software (Version 4.2.3). Packages “TwoSampleMR” and “MRPRESSO” were used for Mendelian randomization analysis. Packages “enrichplot”, “clusterProfiler”and “ggplot2” were used for enrichment analysis and result visualization. *P*_*value*_ < 0.05 was considered to have potential causality.

## Results

### Unvariable MR estimates

**Fig. 2:**
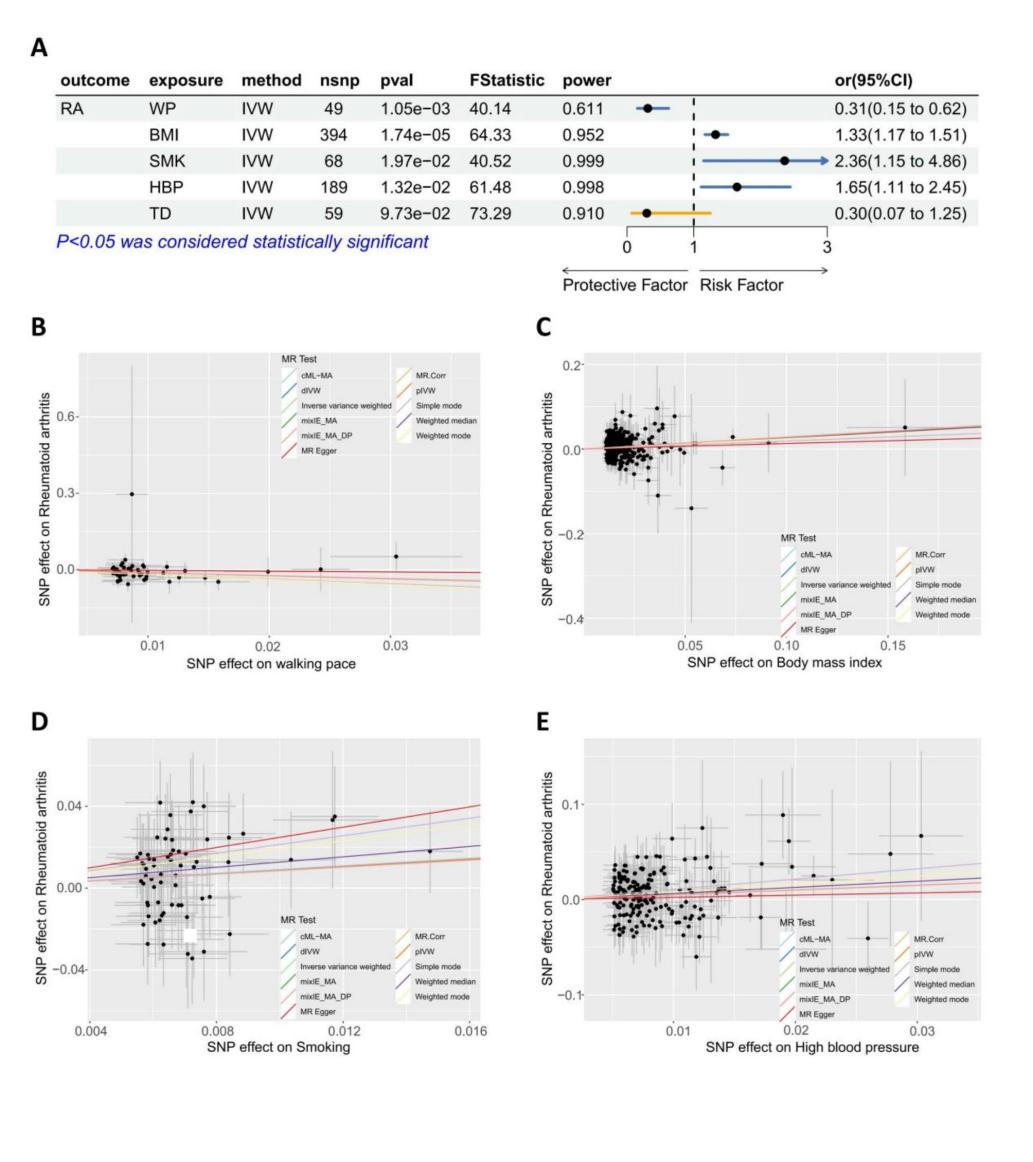
Mendelian randomization results.**A.** Forest plot summarising the overall Mendelian randomization estimates of SNP specificity and the causal effect on RA. **B-E**: Scatter plot of Mendelian randomization analysis for the associations of WP, BMI, SMK, SMK with the risk of RA.

From the GWASs for walking pace (WP), body mass index (BMI), smoking (SMK), high blood pressure (HBP), and type 2 diabetes (TD), we obtained 49, 394, 68, 189, 59 IVs that reached the genome-wide significance level (*p* < 5 × 10^−8^) (**Supplementary Table S4**). Unvariable MR was used to analyze the causal association between WP along with other potential risk factors (BMI, SMK, HBP, TD) with RA. The IVW approach was applied to appraise the causal effect. Results of the MR analysis were expressed as ORs of RA per standard deviation (SD) increase in each factor, as shown in Fig. 2A, indicating a causal relationship between WP (OR, 0.31; 95% CI, 0.15, 0.62; *p* = 1.05 × 10^−3^), BMI (OR, 1.33; 95% CI, 1.17, 1.51; *p* = 1.74 × 10 ^−5^), SMK ( OR, 2.36; 95% CI, 1.15, 4.86; *p* = 1.97 × 10 ^−2^), HBP (OR, 1.65; 95% CI, 1.11, 2.45; *p* = 1.32 × 10 ^−2^), and RA, while no causal relationship was observed between TD (OR, 0.3; 95% CI, 0.07, 1.25; *p* = 9.73 × 10 ^−2^) and RA. Furthermore, BMI, SMK, and HBP showed potential elevations in RA risk, whereas WP exhibited a potential protective effect against RA risk (Fig. 2A). Another cohort (Ncase = 8255, Ncontrol = 409001) was used to verify the protective effect of WP. The replicated MR analysis demonstrated that there was a protective effect of WP on RA (**Supplementary Figure ****S1**,** Supplementary Table S5**). To further validate the results of MR analysis, we employed an additional set of nine methods as supplementary evidence such as weighted mode and simple mode (Fig. 2B-E, **Supplementary Figure S2-6)**. The results demonstrated that WP, BMI, SMK, and HBP still exhibited a causal association with RA among these ten methods. Summary information was presented in **Supplementary Table S4.**

### Multivariable MR-BMA analysis

**Fig. 3:**
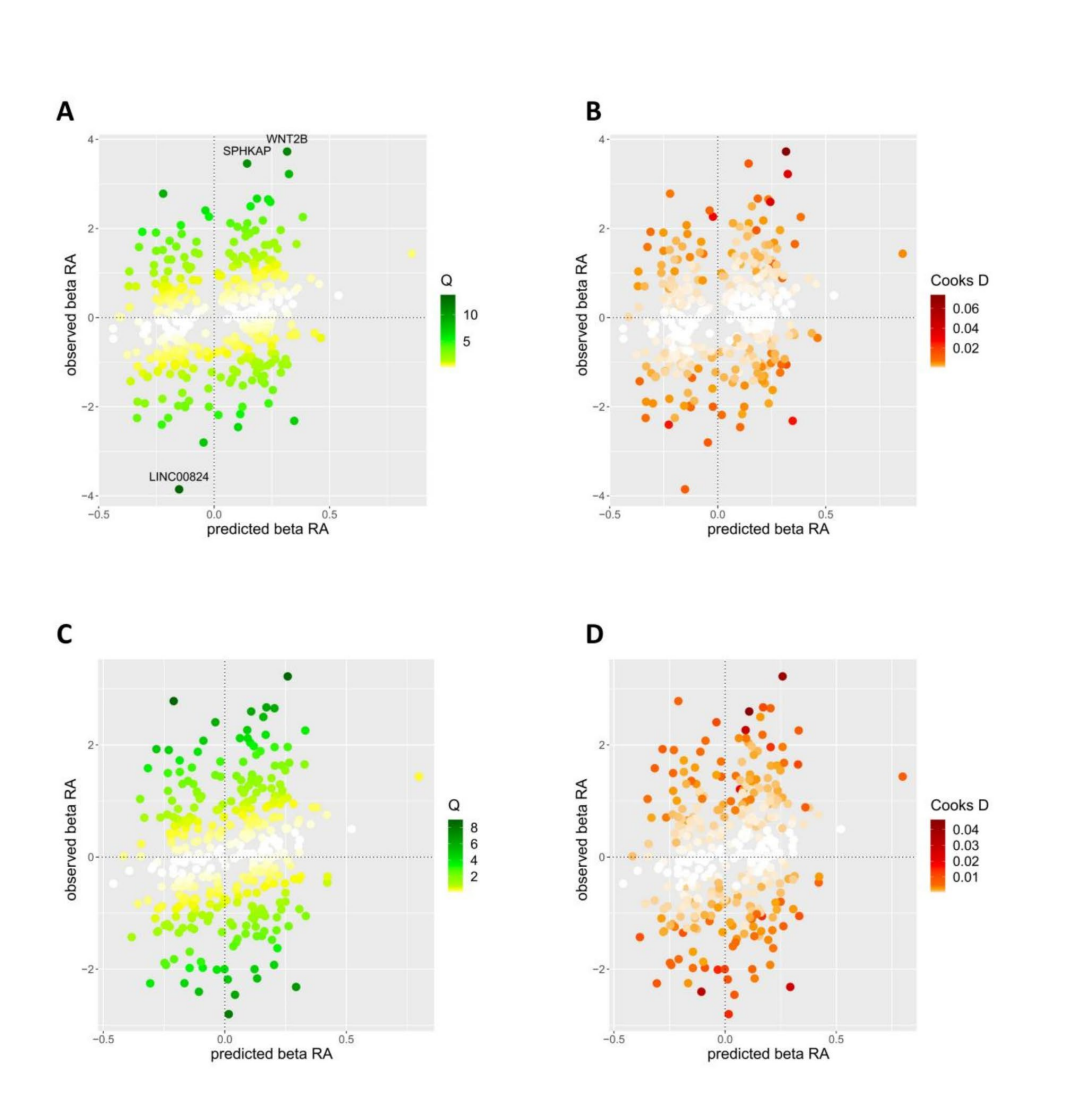
MR-BMA plots for outliers and influential genetic variants.**(A)** Diagnostic plots for invalid SNPs (outliers) **(B)** Diagnostic plots for influential genetic variants **(C)** Diagnostic plots for invalid SNPs after excluding invalid SNPs (rs2894446, rs3790604, rs4605363) **(D)** Diagnostic plots for influential genetic variants after excluding invalid SNPs (rs2894446, rs3790604, rs4605363). When Q-statistic > 10, the instrument for the corresponding gene is invalid and marked with a red box. When Cook’s distance > the threshold, the instrument for the corresponding gene is influential and marked with a red box.

Considering pleiotropy, we employed MR-BMA analysis to evaluate the causal effect between WP and RA to further test the robustness of the results. Three SNPs (SPHKAP, WNT2B, LINC00824) were identified with a Q statistic > 10. In the presence of these SNPs, there was a causal relationship between WP and RA applying MR-BMA analysis (OR, 0.39; 95% CI, 0.17, 0.84; *p* = 1.64 × 10^−2^) (Fig. 3**AB**). However, after excluding them as outliers, the causal relationship between WP and RA remained unchanged (OR, 0.44; 95% CI, 0.20, 0.96; *p* = 3.86 × 10^−2^) (Fig. 3**CD**). The results of Q-statistic and Cook’s distance are shown in Fig. 3 (the dots represented genes corresponding to invalid or influential instruments). Summary information was presented in **Supplementary Table S6-8.**

### Observational study

**Fig. 4:**
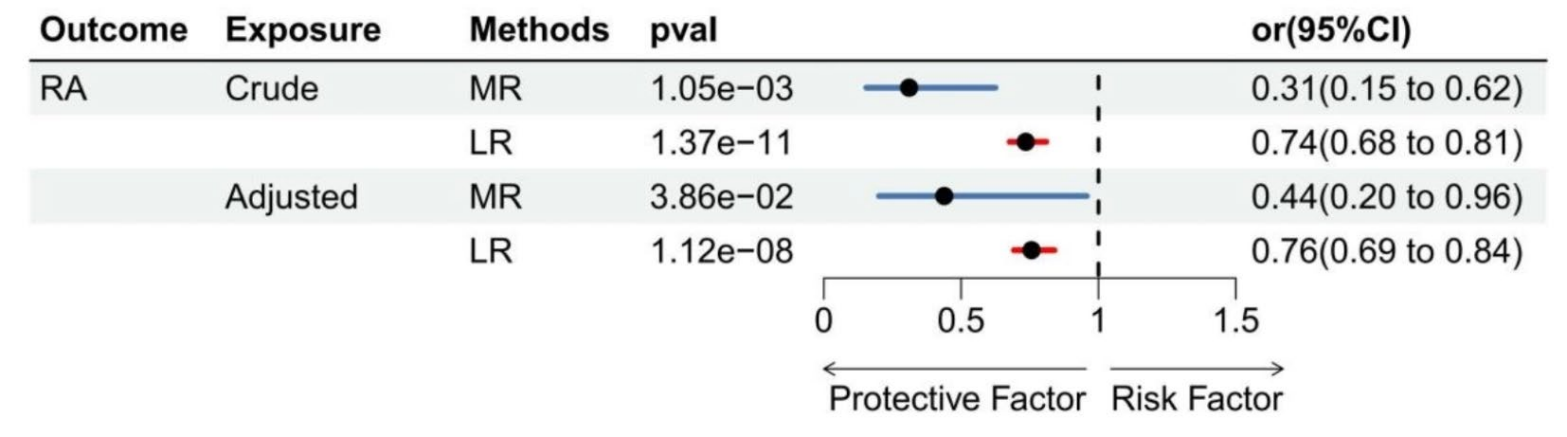
Result of multivariable MR anlysis and observational analysis. Crude: only adjusted for sex and age; Adjusted: adjusted for sex, age, BMI, smoking and high blood pressure; MR: Mendelian randomization; LR: logistic regression

Participants of European individuals in the UK Biobank (*n* = 43,770) including the RA group (*n* = 318) and control group (*n* = 43,452), the specific demographic information, including age, gender, BMI, ever smoked, the percentage of HBP, and WP, was presented in the **Supplementary Table S3**. In the UK Biobank data, WP was categorized into three levels (1: slow, 2: moderate, 3: fast). Initially, the adjustment was performed only for age and sex, and logistic regression was employed to investigate the association, we found that the risk of RA decreased as the WP level increased. Furthermore, additional logistic regression analysis was conducted by incorporating the positive confounding factors (BMI, SMK, HBP) with the MR results. Interestingly, we observed that the conclusion regarding the protective effect of WP on RA remained unchanged even after including these confounding factors (Fig. 4). Summary information was presented in **Supplementary Table S3**,** 9–10**.

### GO and KEGG enrichment analysis

**Fig. 5:**
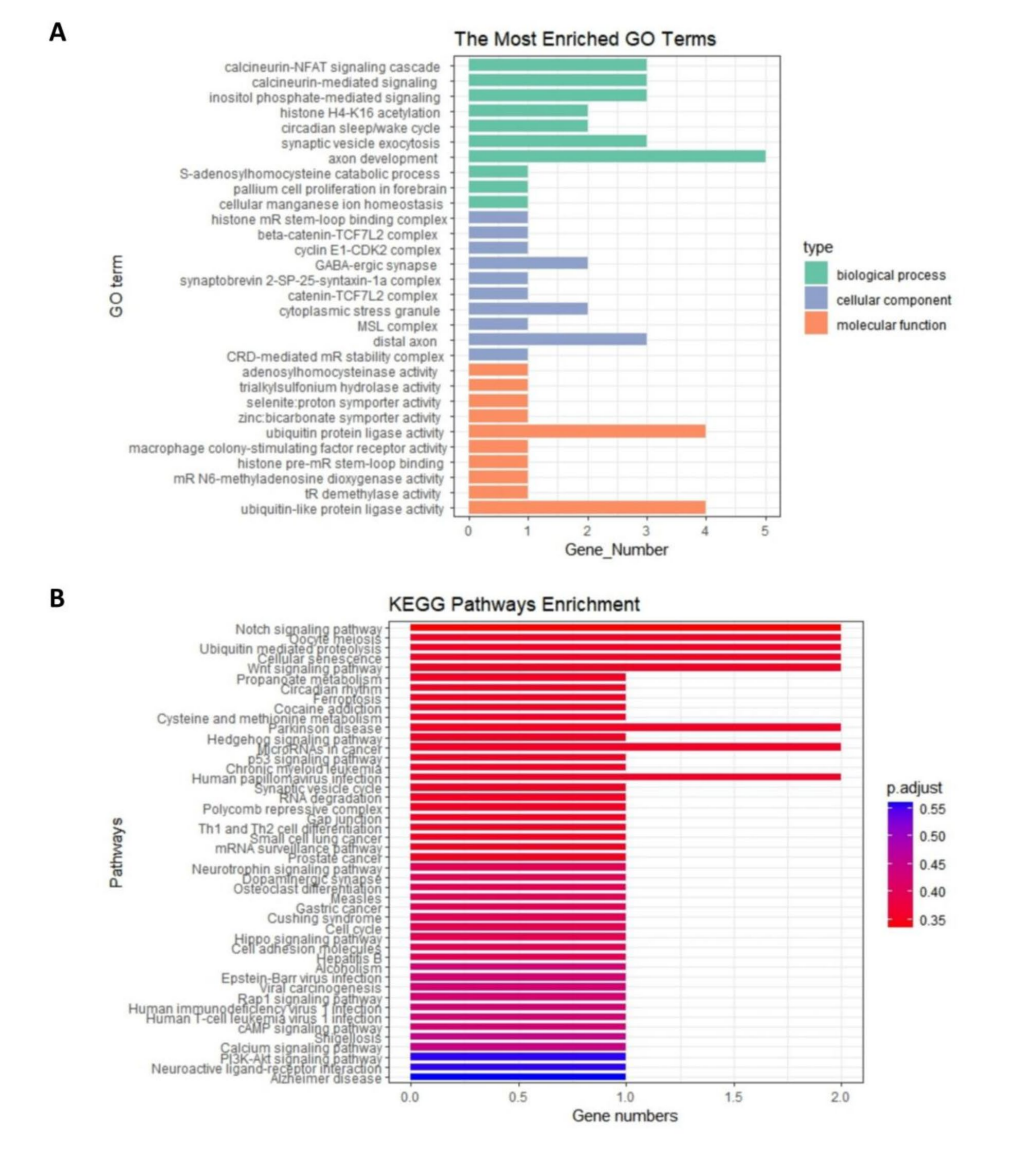
GO enrichment analysis and KEGG enrichment analysis of differential genes where the SNPs obtained by intersecting the summary data of WP and RA.**(A)** The top 10 GO enrichment analyses of biological processes, cellular components, and molecular functions. **(B)** KEGG enrichment analysis of differential genes where the SNPs obtained by intersecting the summary data of WP and RA.

GO terms enrichment analysis of WP on RA found significant enrichment of several crucial regulation pathways. Ten GO biological processes (e.g.,calcineurin-NFAT signaling, cascade calcineurin-mediated signaling, and inositol phosphate-mediated signaling) with *P*_*value*_ < 0.05 were observed to be involved in RA (Fig. 5A). KEGG pathway enrichment analysis with *P*_*value*_ < 0.05 revealed that genes where the SNPs obtained by intersecting WP and RA were primarily enriched in the Notch signaling pathway, Oocyte meiosis, ubiquitin-mediated proteolysis, and cellular senescence (Fig. 5B).

## Discussion

Our study found that WP was inversely associated with the risk of RA after adjusting for age and sex. The association between WP and RA persisted after adjusting for BMI, SMK, and HBP. Then a Mendelian randomization (MR) approach was employed to investigate the potential causal relationship between walking pace (WP) and the risk of developing rheumatoid arthritis (RA). Our findings suggested that walking pace may act as a protective factor against the development of RA.

Previous observational studies have indicated that higher physical activity (PA) levels correlate with reduced risk of RA. Specifically, these studies have demonstrated that PA, such as walking, can effectively reduce the burden of inflammation and improve functional ability^33,34^. Unfortunately, individuals with RA tend to exhibit a preference for sedentary behavior, which may be attributed to factors such as pain, fatigue, or fear of joint damage^35–37^. However, this behavior can lead to the exacerbation of RA-related symptoms, perpetuating a vicious cycle. These findings emphasized the necessity of PA among individuals with RA^38,39^. PA improves RA-related outcomes through various mechanisms, mediated by multiple factors, including the elevated activity of nitric oxide synthase (eNOS), increased blood flow, and the improvement of antioxidant mechanisms^40^. Furthermore, PA ameliorates pain by upregulating the expression of β-endorphin and inducing an anti-inflammatory phenotype^41,42^. In contrast, sun et al. used the MR analyses to suggest physical activity may not help to prevent RA^43^. Considering that PA is a multifaceted trait influenced by numerous genetic variants and environmental factors, it poses challenges in MR analysis due to its complexity. To some extent, it proves the limitations of MR when dealing with highly complex traits. For such complex traits, a multitude of SNPs may be identified, and whether these SNPs can truly represent these complex traits and be used for causal analysis remains debatable. Thus, the focus was narrowed to a specific aspect of physical activity, namely walking pace, to explore the causal relationship between WP and RA from GWAS data in our study.

It is worth noting that our MR analysis accounted for potential confounding factors, including BMI, SMK, HBP, and TD. Moreover, the analysis showed positive correlations between BMI, SMK, and HBP with RA, indicating their potential role as risk factors for the disease, which were consistent with previous studies^44–46^. Surprisingly, MR analysis did not find a causal relationship between TD and RA, similar conclusions were reached by Sun et al., who suggested that the results by MR may be influenced by population distribution^45^.

To reduce the potential impact of confounding factors on the MR estimates of WP and RA, MR-BMA analysis was employed to evaluate the causal effect between WP and RA after adjusting the confounding factors. The MR-BMA plots helped identify outliers and influential genetic variants. By excluding invalid SNPs and influential variants, we ensured the integrity and reliability of our MR analysis, and we found that the causal association between WP and RA remained unchanged regardless of whether outliers were removed or not.

To further explore the biological role of WP on the development of RA, GO enrichment analysis was conducted on the identified genes. GO biological processes (e.g.,calcineurin-NFAT signaling, cascade calcineurin-mediated signaling, and inositol phosphate-mediated signaling) were observed to be involved in RA, which has been supported by previous studies. For example, Park et al. found calcineurin-NFAT1 to 4 pathways are dysregulated in autoimmune diseases^47^, and Gu et al. indicated that the RA patients presented diverse dysfunctions in inositol phosphate metabolism^48^, suggesting that the effect of WP on RA is multifaceted.

While our study provides valuable insights, it is important to acknowledge its limitations. Firstly, gender and age are recognized as important factors influencing the risk of RA development. However, conducting further stratified analysis using publicly available summary-level GWAS data is challenging. In our study, we investigated RA as a whole, as individual patient-level data was not available, precluding subgroup analysis. Secondly, the GWAS summary data used in our study are predominantly focused on individuals of European, which may limit the generalizability of our findings to the whole population. Just as Timmins et al.^49^ conducted a Mendelian Randomization (MR) analysis with a mixed-race population and reached a different conclusion from ours, the LDSC analysis still suggests an association between walking and rheumatoid arthritis. Therefore, the differences in ancestry could be a factor that contributes to varying results to previous studies, potentially introducing bias. Thirdly, our unidirectional MR analysis focused on assessing the causal relationship between walking pace and RA. We did not investigate the two variables’ reverse causality or bidirectional association. Future studies could explore the reciprocal relationship between walking pace and RA to gain a more comprehensive understanding. As we are currently unable to obtain individual genotype data, we cannot conduct one-sample Mendelian Randomization at this time. We will attempt to obtain individual genotype data in the future in order to better confirm the reliability of our conclusions.

## Conclusion

In conclusion, Mendelian randomization design is a valuable approach to studying the causal effects of WP on the risk of RA, our MR study suggested that walking pace acts as a protective factor against the development of rheumatoid arthritis. However further research is needed to fully elucidate the underlying mechanisms involved.

Abbreviations: CI, confidence interval; MR, Mendelian randomization; OR, odds ratio; RA, rheumatoid arthritis; SNP, single- nucleotide polymorphism.

Abbreviations: SNP, single- nucleotide polymorphism; MRA: Bayesian model averaging.

## Electronic supplementary material

Below is the link to the electronic supplementary material.

Supplementary Material 1

## Data Availability

The summary data in our work is publicly accessible. The individual data was obtained from UK Biobank. Data may also be available upon reasonable request to corresponding authors.
