# Supplementary material for: Walking pace is a protective factor for rheumatoid arthritis: a mendelian randomization study
Source: Sci Rep. 2024 Oct 22;14:24886. doi: 10.1038/s41598-024-76666-6 (PMC11496810; doi:10.1038/s41598-024-76666-6)
Supplement: Supplementary file 1 — Supplementary Material 1 [file 41598_2024_76666_MOESM1_ESM.docx]

### Supplementary information

### Walking pace is a protective factor for rheumatoid arthritis: A Mendelian Randomization Study

**Authors**

Qin Zhang^1,2†^, Xiaoxiong Huang^3†^, Yazhong Zhang^4†^, Junzhu Chao^5^, Ruoran Zhou^5^, Roslida Abd Hamid^6^, Yunfang Zhen^7^, Yusheng Li^8^, Cheng Huang^9*^, Wu Xu^2*^, Jun Lin^1,2^*

**Supplementary materials includes:**

**Figure S1-6**

**Table S1 - S10**

**Supplementary Figures**


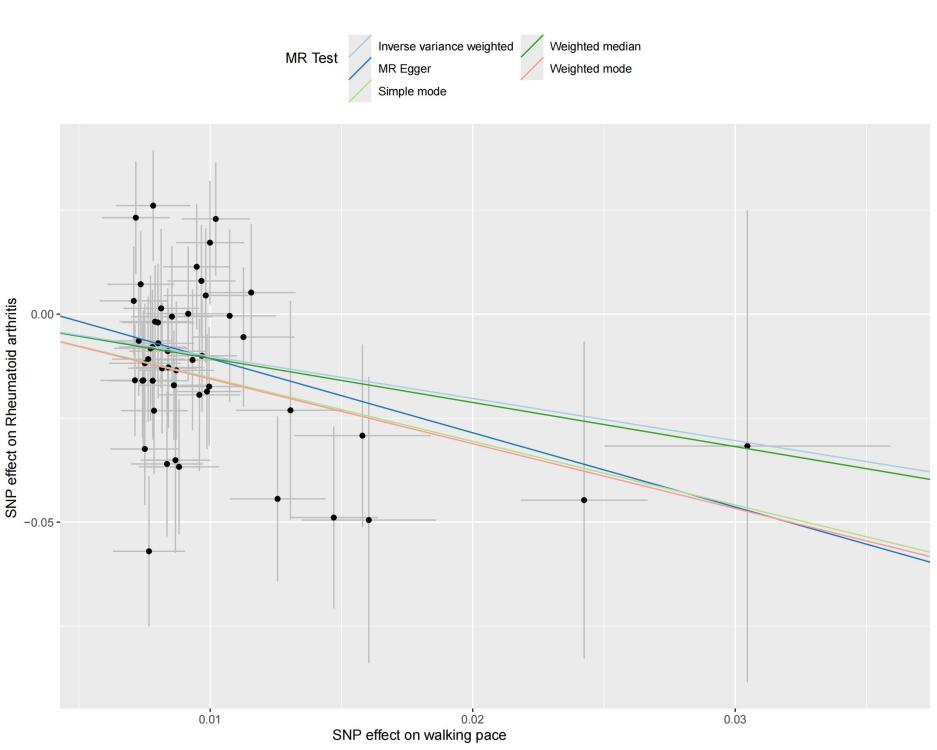


**Figure S1. Scatter plot of replicated MR analysis result.** Five methods includes in the scatter plot of replicated MR analysis result.

**
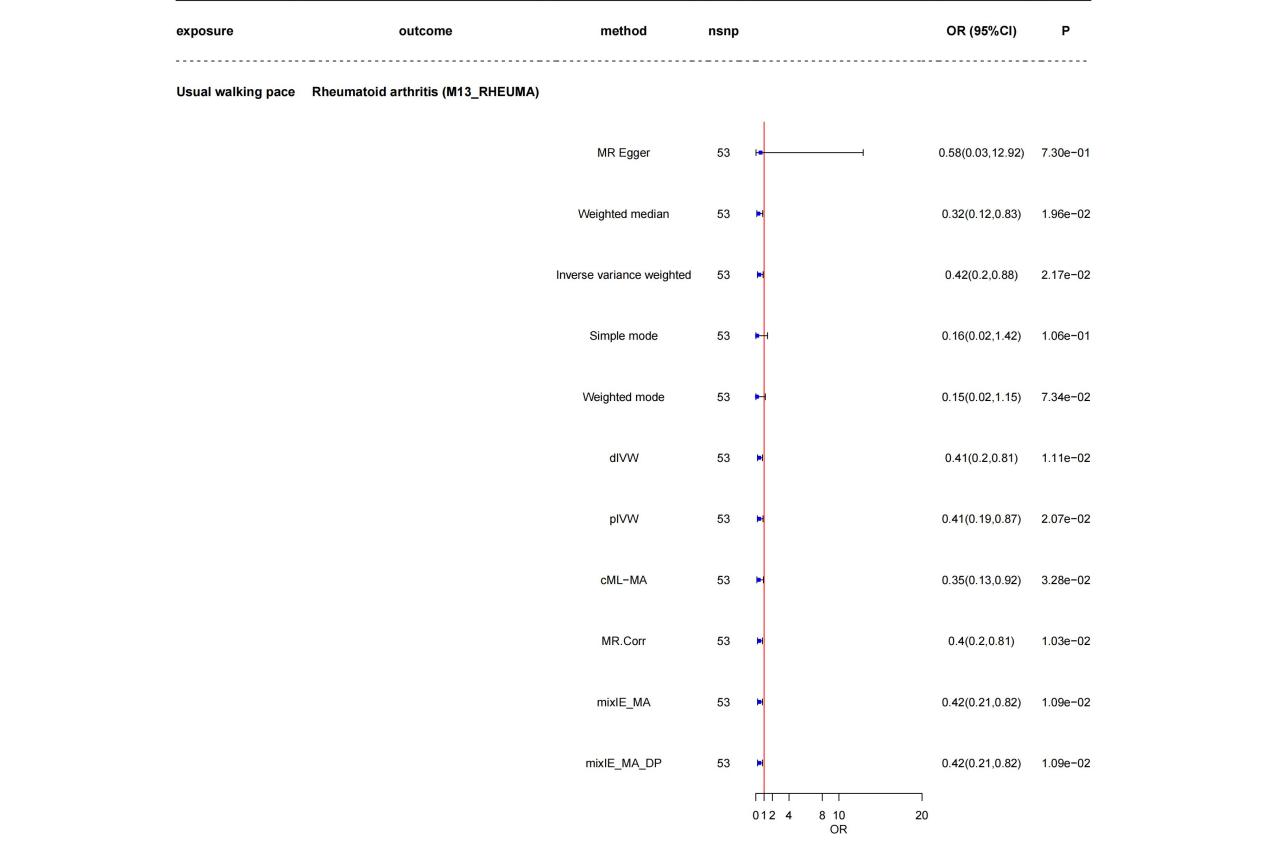
**

**Figure S2. Forest plot of the MR result between Walking pace and RA.**

**
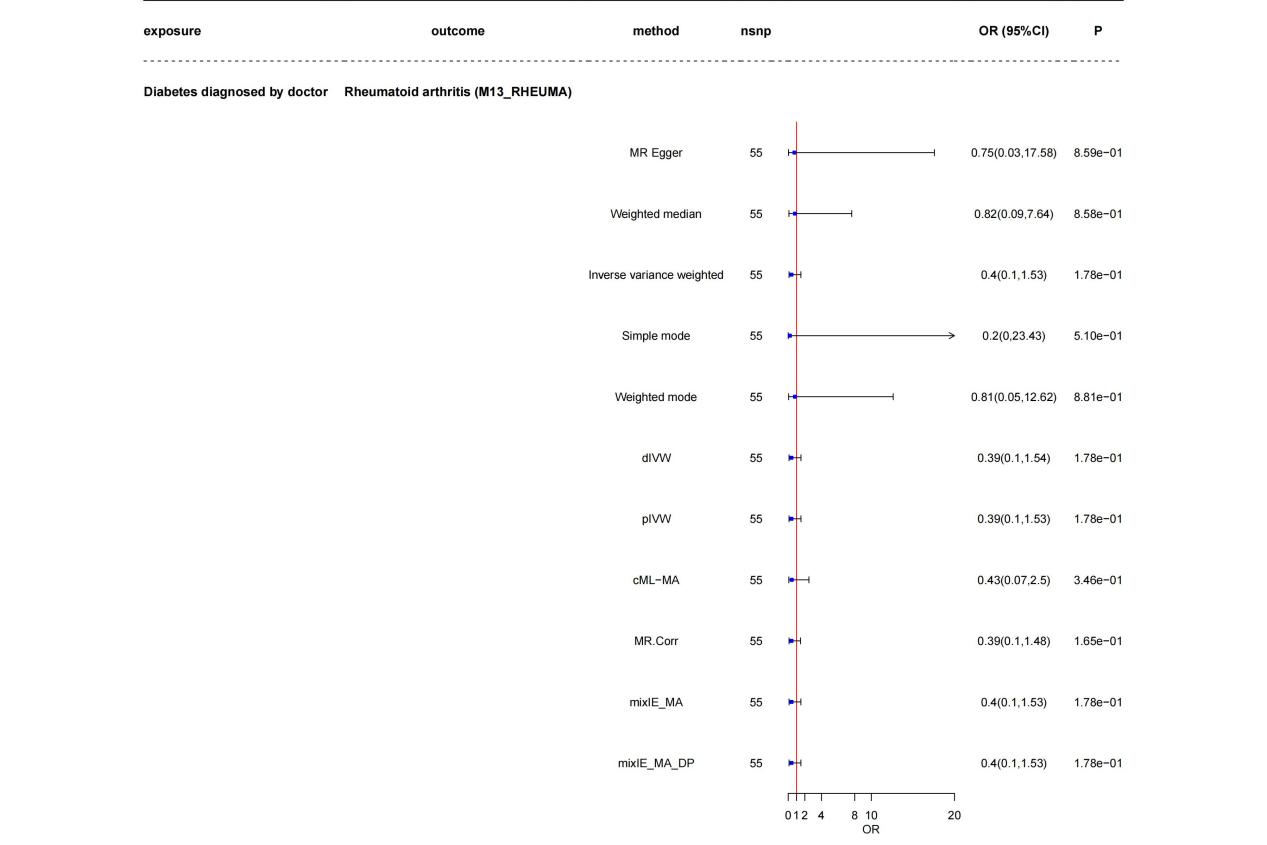
**

**Figure S3.** **Forest plot of the MR result between diabetes and RA.**

**
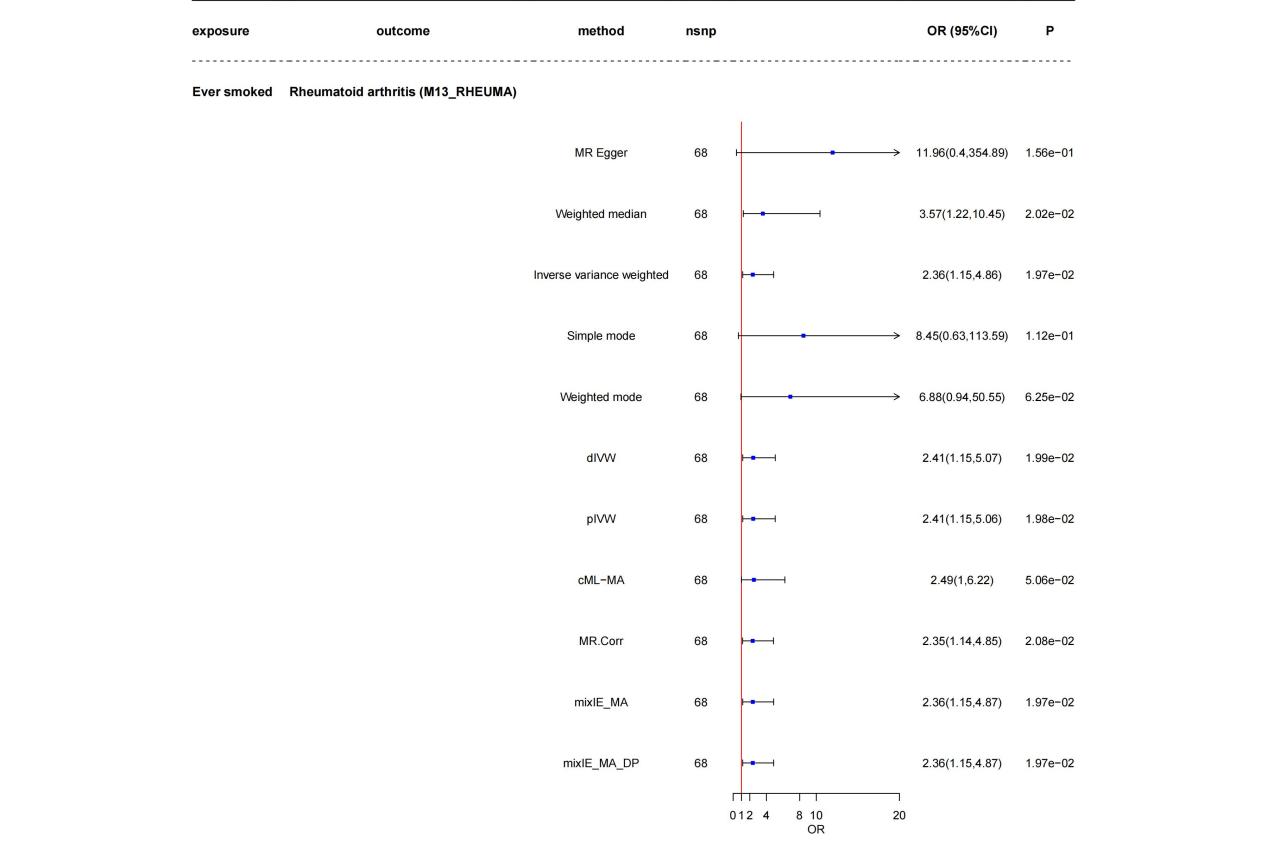
**

**Figure S4. Forest plot of the MR result between ever smoked and RA.**

**
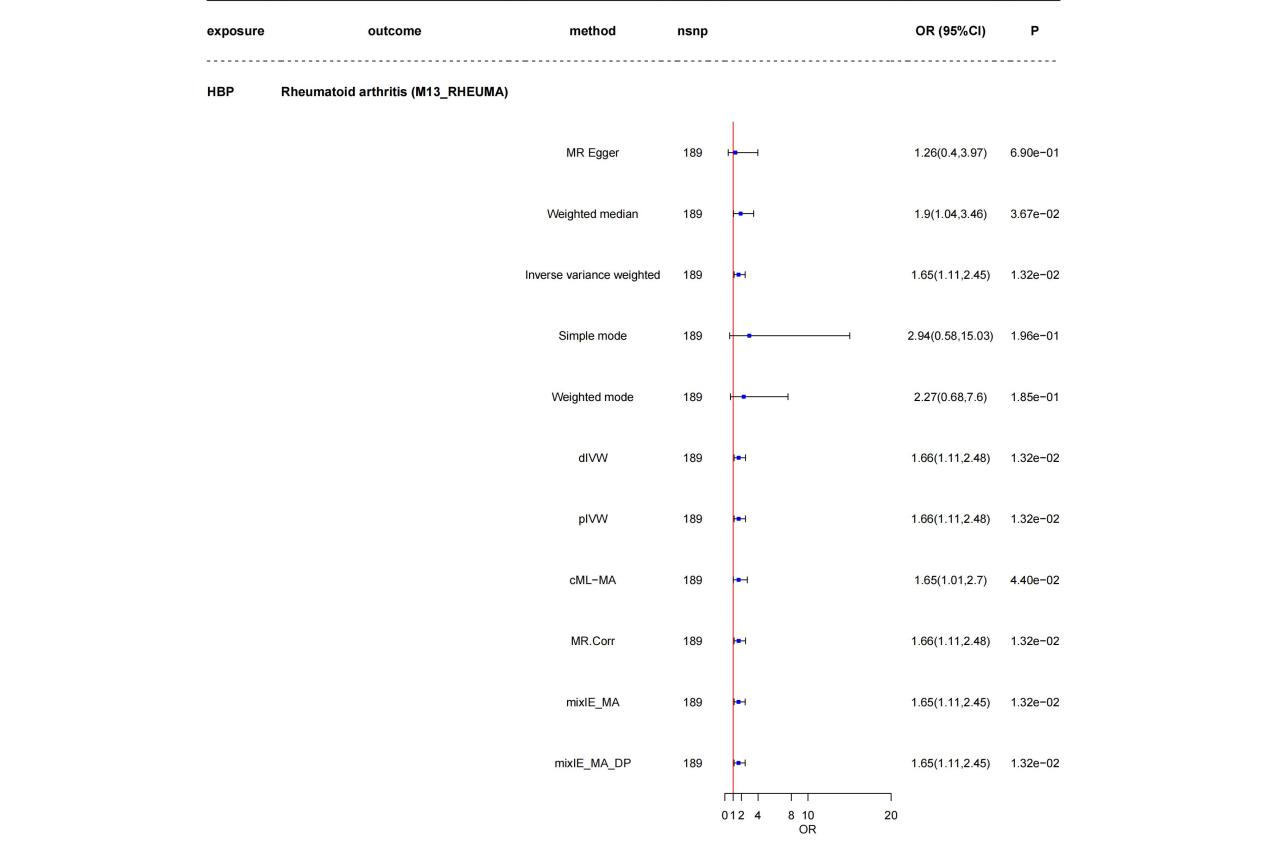
**

**Figure S5. Forest plot of the MR result between high blood pressure and RA.**

**
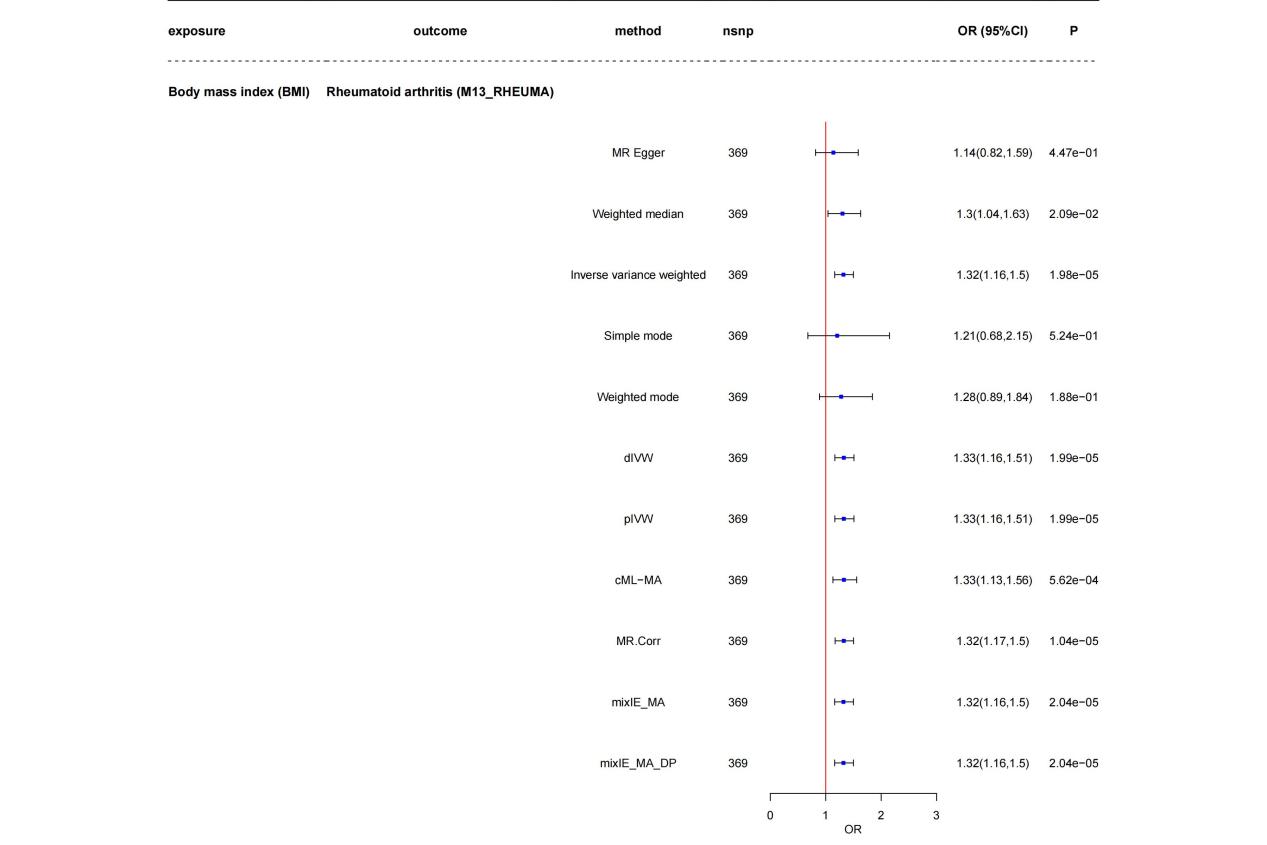
**

**Figure S6. Forest plot of the MR result between BMI and RA.**

**Supplementary Tables**

**Table S1 Characteristics of the data sources.**

| Consortium | MRC-IEU | MRC-IEU | MRC-IEU | MRC-IEU | MRC-IEU | - | - |
| --- | --- | --- | --- | --- | --- | --- | --- |
| Derived from | UKBiobank | UKBiobank | UKBiobank | UKBiobank | UKBiobank | FinnGen | PMID34594039 |
| Unit | SD | SD | SD | SD | SD | - | - |
| Number of SNPs | 9851867 | 9851867 | 9851867 | 9851868 | 9851869 | 16380169 | 24175266 |
| Sample size | 459915 | 461460 | 461066 | 461880 | 461578 | Ncase = 6236, Ncontrol = 147221 | Ncase = 8255, Ncontrol = 409001 |
| Dataset | ukb-b-4711 | ukb-b-19953 | ukb-b-20261 | ukb-b-14177 | ukb-b-10753 | finn-b-M13_RHEUMA | ebi-a-GCST90018910 |
| Data Source | IEU Open GWAS project | IEU Open GWAS project | IEU Open GWAS project | IEU Open GWAS project | IEU Open GWAS project | IEU Open GWAS project | IEU Open GWAS project |
| Population | European | European | European | European | European | European | European |
| Phenotype | Walking pace | Body mass index (BMI) | Ever smoked | High blood pressure | Diabetes diagnosed by doctor | Rheumatoid arthritis | Rheumatoid arthritis |

**Table S2 The information about harmonized SNPs used for MR analysis**

| **snp** | **chr** | **pos_hg19** | **pos_hg38** | **hgnc** |
| --- | --- | --- | --- | --- |
| rs10750025 | 11 | 113424042 | 113553320 | DRD2 |
| rs10828258 | 10 | 21929734 | 21640805 | MLLT10 |
| rs10862220 | 12 | 81430599 | 81036820 | ACSS3 |
| rs10883618 | 10 | 103117653 | 101357896 | BTRC |
| rs11039324 | 11 | 47665686 | 47644134 | MTCH2 |
| rs11077815 | 17 | 74389890 | 76393809 | UBE2O |
| rs11150623 | 16 | 28881001 | 28869680 | SH2B1 |
| rs11152989 | 6 | 96936061 | 96488185 | UFL1-AS1 |
| rs113825410 | 1 | 40057543 | 39591871 | PABPC4 |
| rs11548200 | 1 | 156290656 | 156320865 | CCT3 |
| rs11682482 | 2 | 226486479 | 225621763 | NYAP2 |
| rs11732213 | 4 | 1704244 | 1702517 | SLBP |
| rs11761141 | 7 | 69423362 | 69958376 | AUTS2 |
| rs11848096 | 14 | 100969235 | 100502898 | WDR25 |
| rs12042959 | 1 | 243533273 | 243369971 | SDCCAG8 |
| rs12461902 | 19 | 30265235 | 29774328 | CCNE1 |
| rs12883788 | 14 | 33303540 | 32834334 | AKAP6 |
| rs13107325 | 4 | 103188709 | 102267552 | SLC39A8 |
| rs144333966 | 4 | 61109385 | 60243667 | DPP3P1 |
| rs1592 | 2 | 135722143 | 134964573 | MAP3K19 |
| rs2037735 | 19 | 48012469 | 47509212 | NAPA |
| rs205262 | 6 | 34563164 | 34595387 | C6orf106 |
| rs2170670 | 12 | 16944621 | 16791687 | RP11-239A17.1 |
| rs2280406 | 3 | 49941436 | 49904003 | MST1R |
| rs2297600 | 1 | 32207581 | 31741980 | ADGRB2 |
| rs2439823 | 10 | 99778226 | 98018469 | CRTAC1 |
| rs2602731 | 19 | 4944771 | 4944759 | UHRF1 |
| rs2645979 | 12 | 84017043 | 83623264 | RP11-384P14.1 |
| rs273512 | 19 | 18224729 | 18113919 | MAST3 |
| rs28519617 | 3 | 135874930 | 136156088 | MSL2 |
| rs35711462 | 5 | 50847577 | 51551743 | AC116606.1 |
| rs4109292 | 10 | 126710654 | 125022085 | CTBP2 |
| rs4516268 | 17 | 1846831 | 1943537 | RTN4RL1 |
| rs4643373 | 17 | 47123423 | 49046061 | IGF2BP1 |
| rs4715208 | 6 | 50829471 | 50861758 | RPS17P5 |
| rs4839898 | 6 | 97546759 | 97098883 | KLHL32 |
| rs55680124 | 2 | 105984624 | 105368167 | FHL2 |
| rs57800857 | 4 | 140863365 | 139942211 | MAML3 |
| rs613872 | 18 | 53210302 | 55543071 | TCF4 |
| rs62048402 | 16 | 53803223 | 53769311 | FTO |
| rs6763292 | 3 | 129044705 | 129325862 | H1FX-AS1 |
| rs7789719 | 7 | 66893916 | 67428929 | AC006480.1 |
| rs8010773 | 14 | 46956863 | 46487660 | LINC00871 |
| rs8011870 | 14 | 80173397 | 79707054 | NRXN3 |
| rs819167 | 20 | 32903687 | 34315881 | AHCY |
| rs830627 | 3 | 71675270 | 71626119 | RP11-154H23.4 |
| rs891387 | 18 | 21103909 | 23523945 | C18orf8 |
| rs9366651 | 6 | 26336696 | 26336468 | ZFP57 |
| rs9783304 | 11 | 43660255 | 43638705 | RP11-472I20.4 |

**Table S3 The data of UK Biobank**

| **Variable** | **RA (N=318)** | **Non_RA (N=43452)** | **Pval** |
| --- | --- | --- | --- |
| Age (M±SD) | 56.64 ± 7.31 | 55.16 ± 7.59 | 3.557E-04 |
| BMI(M±SD) | 27.44 ± 4.78 | 26.68 ± 4.28 | 4.893E-03 |
| Sex (percentage) |  |  | 3.674E-09 |
| Male | 31.9 | 48.6 |  |
| Female | 68.1 | 51.4 |  |
| Ever smoked (percentage) |  |  | 4.629E-02 |
| Ever | 37.5 | 33.3 |  |
| Never | 62.5 | 66.7 |  |
| HBP (percentage) |  |  | 2.364E-01 |
| Yes | 55.3 | 51.8 |  |
| No | 44.7 | 48.2 |  |
| Walking pace |  |  | 3.927E-06 |
| Slow | 11.3 | 3.1 |  |
| Medium | 51.9 | 47.7 |  |
| High | 36.8 | 49.2 |  |

**Table S4 Mendelian randomization results**

| **outcome** | Rheumatoid arthritis | Rheumatoid arthritis | Rheumatoid arthritis | Rheumatoid arthritis | Rheumatoid arthritis | Rheumatoid arthritis | Rheumatoid arthritis | Rheumatoid arthritis | Rheumatoid arthritis | Rheumatoid arthritis | Rheumatoid arthritis | Rheumatoid arthritis | Rheumatoid arthritis | Rheumatoid arthritis | Rheumatoid arthritis | Rheumatoid arthritis | Rheumatoid arthritis | Rheumatoid arthritis | Rheumatoid arthritis | Rheumatoid arthritis | Rheumatoid arthritis | Rheumatoid arthritis | Rheumatoid arthritis | Rheumatoid arthritis | Rheumatoid arthritis |
| --- | --- | --- | --- | --- | --- | --- | --- | --- | --- | --- | --- | --- | --- | --- | --- | --- | --- | --- | --- | --- | --- | --- | --- | --- | --- |
| **exposure** | Walking pace | Walking pace | Walking pace | Walking pace | Walking pace | Body mass index | Body mass index | Body mass index | Body mass index | Body mass index | High blood pressure | High blood pressure | High blood pressure | High blood pressure | High blood pressure | Ever smoked | Ever smoked | Ever smoked | Ever smoked | Ever smoked | Diabetes | Diabetes | Diabetes | Diabetes | Diabetes |
| **method** | MR Egger | Weighted median | Inverse variance weighted | Simple mode | Weighted mode | MR Egger | Weighted median | Inverse variance weighted | Simple mode | Weighted mode | MR Egger | Weighted median | Inverse variance weighted | Simple mode | Weighted mode | MR Egger | Weighted median | Inverse variance weighted | Simple mode | Weighted mode | MR Egger | Weighted median | Inverse variance weighted | Simple mode | Weighted mode |
| **nsnp** | 49 | 49 | 49 | 49 | 49 | 394 | 394 | 394 | 394 | 394 | 189 | 189 | 189 | 189 | 189 | 68 | 68 | 68 | 68 | 68 | 59 | 59 | 59 | 59 | 59 |
| **b** | -0.304359719 | -1.212304433 | -1.169383257 | -1.842624877 | -1.934112426 | 0.20730552 | 0.264412184 | 0.282419231 | 0.180837242 | 0.247763151 | 0.233666671 | 0.640792198 | 0.499308022 | 1.07949074 | 0.820258218 | 2.481905166 | 1.272488027 | 0.859458029 | 2.134730033 | 1.928109565 | -0.613688734 | -0.306326913 | -1.219804043 | -1.856706438 | -0.191206453 |
| **se** | 1.463817763 | 0.491783543 | 0.356735269 | 1.154844427 | 1.036019145 | 0.174355151 | 0.115793964 | 0.065745626 | 0.301088082 | 0.175103239 | 0.584161901 | 0.306660554 | 0.201519435 | 0.831865977 | 0.615991558 | 1.729549322 | 0.547844087 | 0.36860412 | 1.325443835 | 1.017765898 | 1.720094582 | 1.088335968 | 0.735581922 | 2.386567458 | 1.376298599 |
| **pval** | 8.362E-01 | 1.370E-02 | 1.045E-03 | 1.172E-01 | 6.803E-02 | 2.352E-01 | 2.240E-02 | 1.742E-05 | 5.484E-01 | 1.579E-01 | 6.896E-01 | 3.666E-02 | 1.322E-02 | 1.960E-01 | 1.846E-01 | 1.560E-01 | 2.019E-02 | 1.972E-02 | 1.120E-01 | 6.248E-02 | 7.226E-01 | 7.784E-01 | 9.726E-02 | 4.397E-01 | 8.900E-01 |
| **lo_ci** | -3.173442534 | -2.176200177 | -1.868584384 | -4.106119953 | -3.964709951 | -0.134430575 | 0.037456015 | 0.153557805 | -0.4092954 | -0.095439199 | -0.911290654 | 0.039737511 | 0.104329928 | -0.550966574 | -0.387085235 | -0.908011505 | 0.198713616 | 0.136993954 | -0.463139883 | -0.066711594 | -3.985074115 | -2.439465411 | -2.661544611 | -6.534378656 | -2.888751707 |
| **up_ci** | 2.564723097 | -0.24840869 | -0.470182129 | 0.420870199 | 0.096485099 | 0.549041615 | 0.491368354 | 0.411280658 | 0.770969883 | 0.5909655 | 1.378623996 | 1.241846885 | 0.894286115 | 2.709948054 | 2.027601671 | 5.871821837 | 2.346262437 | 1.581922105 | 4.732599949 | 3.922930724 | 2.757696647 | 1.826811585 | 0.221936525 | 2.82096578 | 2.506338801 |
| **or** | 0.737595492 | 0.297510895 | 0.310558417 | 0.158401097 | 0.144552513 | 1.230358414 | 1.302665024 | 1.326334645 | 1.198220143 | 1.281156455 | 1.263223353 | 1.897983863 | 1.647580786 | 2.943180326 | 2.271086196 | 11.96403619 | 3.569723089 | 2.361880277 | 8.454763695 | 6.876498373 | 0.541350284 | 0.736145925 | 0.295288025 | 0.156186193 | 0.825962048 |
| **or_lci95** | 0.041859248 | 0.113471886 | 0.154341996 | 0.016471561 | 0.018973539 | 0.874213569 | 1.038166332 | 1.165975185 | 0.664118023 | 0.908973627 | 0.402005039 | 1.040537609 | 1.109966604 | 0.576392415 | 0.679033215 | 0.403325438 | 1.219832575 | 1.146821215 | 0.629304598 | 0.935464956 | 0.018591066 | 0.087207459 | 0.069840262 | 0.001452631 | 0.055645631 |
| **or_uci95** | 12.99705894 | 0.780041083 | 0.624888448 | 1.523286542 | 1.101293171 | 1.731592691 | 1.634551335 | 1.508748739 | 2.16186199 | 1.805731008 | 3.96943591 | 3.462001481 | 2.445589296 | 15.02849483 | 7.595847154 | 354.8949524 | 10.4464524 | 4.864296519 | 113.5905083 | 50.54837124 | 15.76349221 | 6.214042094 | 1.248492128 | 16.79306125 | 12.25996163 |
| **FStatistic** | 40.14 |  |  |  |  | 64.33 |  |  |  |  | 61.48 |  |  |  |  | 40.52 |  |  |  |  | 73.29 |  |  |  |  |
| **power** | 0.611255302 |  |  |  |  | 0.951528107 |  |  |  |  | 0.997798241 |  |  |  |  | 0.999481179 |  |  |  |  | 0.909595636 |  |  |  |  |
| **Egger_Heterogeneity pval** | 0.7365356 |  |  |  |  | 0.9966062 |  |  |  |  | 0.9698033 |  |  |  |  | 0.9298784 |  |  |  |  | 0.5513401 |  |  |  |  |
| **Egger_intercept pval** | 0.5452552 |  |  |  |  | 0.3433759 |  |  |  |  | 0.6286122 |  |  |  |  | 0.3404924 |  |  |  |  | 0.6617128 |  |  |  |  |

**Table S5. The result of replicated MR analysis result**

| **exposure** | | **outcome** | **method** | **nsnp** | **b** | **se** | **pval** |
| --- | --- | --- | --- | --- | --- | --- | --- |
| Walking pace | Rheumatoid arthritis | | MR Egger | 56 | -1.430307117 | 1.099757043 | 0.198930037 |
| Walking pace | Rheumatoid arthritis | | Weighted median | 56 | -1.119001215 | 0.326187886 | 0.000602377 |
| Walking pace | Rheumatoid arthritis | | Inverse variance weighted | 56 | -1.035691091 | 0.248831226 | 3.15E-05 |
| Walking pace | Rheumatoid arthritis | | Simple mode | 56 | -1.616028825 | 0.799959553 | 0.048250431 |
| Walking pace | Rheumatoid arthritis | | Weighted mode | 56 | -1.644035255 | 0.812033245 | 0.047775771 |

**Table S6. Results of MR-BMA**

| **rf** | **se** | **OR** | **lower_limit** | **upper_limit** | **p** |
| --- | --- | --- | --- | --- | --- |
| BMI | -0.091729251 | 1.295145483 | 1.550244039 | 1.082024365 | 4.811E-03 |
| WP | 0.395660434 | 0.38686532 | 0.178142211 | 0.840142124 | 1.638E-02 |
| HBP | -0.306522213 | 1.960386549 | 3.574857124 | 1.075040285 | 2.809E-02 |
| SMK | -0.699243375 | 2.736190881 | 10.77343928 | 0.694925765 | 1.500E-01 |

**Table S7. invalid SNPs**

| **rs** | **genes** | **maxQ** |
| --- | --- | --- |
| rs2894446 | LINC00824 | 14.411 |
| rs3790604 | WNT2B | 13.723 |
| rs4605363 | SPHKAP | 11.298 |

**Table S8 Results after excluding invalid SNPs**

| **rf** | **se** | **OR** | **lower_limit** | **upper_limit** | **p** |
| --- | --- | --- | --- | --- | --- |
| BMI | -0.089192415 | 1.270166313 | 1.512804149 | 1.066445029 | 7.335E-03 |
| WP | 0.39859114 | 0.438471687 | 0.200749248 | 0.95769933 | 3.860E-02 |
| SMK | -0.61774447 | 2.936439025 | 9.854978826 | 0.874956131 | 8.120E-02 |
| HBP | -0.358321018 | 1.643457001 | 3.317167133 | 0.814234197 | 1.656E-01 |

**Table S9 Results after the adjusted for sex and age**

|  | **OR** | **CI_lower** | **CI_upper** | **pvalue** |
| --- | --- | --- | --- | --- |
| (Intercept) | 0.004006352 | 0.00162625 | 0.009644695 | 4.996E-34 |
| wp | 0.735414958 | 0.676793335 | 0.810045979 | 1.375E-11 |
| Age | 1.02948639 | 1.014135725 | 1.045276667 | 1.644E-04 |
| Sex | 0.481545879 | 0.378460026 | 0.608696502 | 1.590E-09 |

**Table S10 Results after the adjusted for sex, age, BMI, SMK and HBP**

|  | **OR** | **CI_lower** | **CI_upper** | **pvalue** |
| --- | --- | --- | --- | --- |
| (Intercept) | 0.00170274 | 0.00053891 | 0.005270456 | 5.628E-28 |
| WP | 0.756336898 | 0.692031935 | 0.839622703 | 1.117E-08 |
| Age | 1.027764456 | 1.011964334 | 1.044006227 | 5.703E-04 |
| Sex | 0.456042929 | 0.357642189 | 0.577773103 | 1.327E-10 |
| BMI | 1.02858423 | 1.004338526 | 1.052480774 | 1.827E-02 |
| Ever smoked | 1.237887866 | 1.040515191 | 1.467534555 | 1.498E-02 |
| HBP | 1.072303815 | 0.852325751 | 1.351444005 | 5.524E-01 |
